# “It hurt but it helped”: A mixed methods audit of the implementation of trauma- focused cognitive-behavioral therapy for psychosis

**DOI:** 10.3389/fpsyt.2022.946615

**Published:** 2022-10-13

**Authors:** Amy Hardy, Sophie Good, Jayde Dix, Eleanor Longden

**Affiliations:** ^1^Institute of Psychiatry, Psychology and Neuroscience, King’s College London, London, United Kingdom; ^2^South London and Maudsley NHS Foundation Trust, London, United Kingdom; ^3^North East London NHS Foundation Trust, London, United Kingdom; ^4^Psychosis Research Unit, Greater Manchester Mental Health NHS Foundation Trust, Manchester, United Kingdom; ^5^Division of Psychology and Mental Health, School of Health Sciences, Faculty of Biology, Medicine and Health, Manchester Academic Health Science Centre, The University of Manchester, Manchester, United Kingdom; ^6^Complex Trauma and Resilience Research Unit, Greater Manchester Mental Health NHS Foundation Trust, Manchester, United Kingdom

**Keywords:** trauma, psychosis, post-traumatic stress, cognitive-behavioral therapy, trauma therapy

## Abstract

**Background:**

Emerging evidence supports the safety, acceptability, and efficacy of trauma therapies for people experiencing post-traumatic stress and psychosis, despite common concerns about iatrogenic harm when processing trauma memories for this population. However, to date there have been no mixed-method studies examining whether trauma-focused therapy can be implemented in routine care. This study reports an audit of a post-traumatic stress in psychosis clinic based in an inner-city trust in the U.K. National Health Service.

**Materials and methods:**

People under the care of psychosis community mental health teams with a significant history of past trauma were referred to the clinic by their multidisciplinary clinicians. Referral outcomes were recorded, including the proportion of people for whom trauma-focused cognitive-behavior therapy for psychosis was indicated. Post-traumatic stress symptoms were assessed pre- and post-therapy for clinically significant change on the Post-traumatic Stress Checklist (version 4) and Post-traumatic Stress Checklist (version 5). A subgroup of service users was also interviewed about their experience of therapy, with transcripts analyzed using inductive thematic analysis.

**Results:**

Seventy one service-users were referred to the clinic between 2014 and 2018, of which 51 (71.8%) attended an assessment. Of these, 20 (39.2%) were identified as having clinically significant PTSD symptoms with re-experiencing and were offered trauma-focused cognitive-behavior therapy for psychosis. Sixteen (80%) accepted and completed therapy, with no dropouts, and received a mean of 17.54 sessions (SD = 17.60, range = 12–91). There were no serious adverse events related to therapy. Clinically significant change was observed in 68.8% (*n* = 11) of the therapy group and post-therapy six people (37.5%) no longer met the threshold for clinically significant PTSD. Six service users completed an interview about their therapy experiences with findings organized within four main themes and associated subthemes: (1) Perseverance, (2) Establishing safety, (3) The challenges of therapy, and (4) Rebuilding one’s life after trauma.

**Conclusion:**

Trauma-focused cognitive-behavior therapy for psychosis can be safe, acceptable, and effective when implemented in routine care. Lived experience perspectives highlight the emotional demands of therapy and long-term impact of trauma, thus underscoring the necessity of sufficient support and continuity of care both during and after therapy.

## Introduction

While traumatic events are common in society, communities at the intersection of socioeconomic inequalities experience them at higher rates (1, 2). People with psychosis constitute one of these groups, with trauma also associated with worse treatment response and outcomes (3–6). Accordingly, rates of current Post-Traumatic Stress Disorder (PTSD) are approximately five times higher in people with psychosis (15%) compared to the general population, with a greater proportion experiencing subclinical PTSD symptoms and other trauma reactions (7, 8). Childhood victimization has replicable, prospective, dose-response and reversible associations with psychosis, with evidence of mediation by psychological mechanisms, supporting a potential causal role (9–11). Despite the marked impact of trauma on the lives of people with psychosis, they face significant challenges in accessing evidence-based therapies. Routine enquiry about trauma and its effects is lacking, and psychosis is often an exclusion criterion for trauma therapy trials and traumatic stress services (12, 13). Research suggests this exclusion is partly due to clinician’ anxieties about people with distressing psychosis not being sufficiently able to tolerate processing of episodic memories (14). Trauma memory processing is hypothesized to be a key mechanism of change in trauma-focused therapies for PTSD and is emotionally burdensome, leading to concerns about its potential for iatrogenic harm in psychosis (15).

However, encouraging findings from case series, feasibility studies and randomized controlled trials suggest that trauma-focused therapies that include memory processing are safe, acceptable, and efficacious for PTSD symptoms in psychosis (16–23). In line with the Medical Research Council’s recommendations for developing complex interventions, implementation should now be investigated (24). This is an important step as efficacious interventions may not generalize to real world settings and can face other barriers to implementation outside of a trial context, where real world complexity is necessarily constrained for the purposes of evaluation (25). This audit study therefore reports a mixed-methods analysis of therapy implementation in a post-traumatic stress in psychosis (PTSp) clinic based in an inner-city trust in the U.K. National Health Service (NHS). In the U.K, the National Institute for Health and Care Excellence (26) guidelines for psychosis and schizophrenia recommend that trauma and its consequences are routinely assessed, particularly in early intervention and, if clinically significant trauma effects are identified, to follow their guidelines for PTSD. The PTSD guidance specifies using trauma-focused cognitive-behavioral therapy (tf-CBT) or Eye Movement and Desensitization Reprocessing (EMDR) therapy for the treatment of PTSD, with tf-CBT including Prolonged Exposure (PE), Narrative Exposure Therapy (NET), Cognitive Processing Therapy (CPT) and Cognitive Therapy (CT) (27). It is recognized that for complex needs, additional emphasis should be placed on addressing the therapeutic relationship, barriers to therapy, and social circumstances, in relation to therapy content, process, and duration.

This NICE guidance provides a helpful mandate for UK therapists to implement trauma therapies involving memory processing for psychosis. However, an implementation dilemma is that it is specific to PTSD and does not provide recommendations for how to intervene with the range of other, common trauma reactions people may experience, including trauma-related psychosis. We propose that the NICE recommended individual psychological intervention for distressing psychotic experiences, cognitive-behavioral therapy for psychosis (CBTp), can provide a helpful framework for clinicians in this regard. Whilst there is marked overlap between CBTp and tf-CBT (e.g., managing emotions, evaluation of thoughts and working toward valued goals), their key distinguishing feature is that the former addresses the sensory-perceptual experiences that drive hallucinations (such as voices, visions and sensations) and unusual beliefs, whereas the latter tends to emphasize the processing of intrusive episodic memories to ameliorate re-experiencing, the hallmark symptom of PTSD (characterized by vivid, intrusive flashbacks and nightmares, that are accompanied by a sense of the traumatic experience happening again in the “here and now”). CBTp addresses trauma consequences through collaborative development of an understanding of how trauma impacts sensory-perceptual experiences, appraisals, and their behavioral and emotional consequences, providing a validating and normalizing account which may reduce distress (28, 29). Cognitive and behavioral techniques can then be employed to modify trauma-related meanings and related coping strategies. CBTp therefore provides a means to support people and their clinicians in addressing trauma-related effects, including hallucinations and unusual beliefs, occurring in the absence of re-experiencing.

A further challenge in implementing therapy to address trauma in psychosis is whether, and how, to integrate tf-CBT and CBTp for people who have both re-experiencing and psychosis. Consecutive delivery of targeted therapies is a possibility and has been researched in a US context (9, 30). However, it may not be optimally acceptable nor effective to treat PTSD and psychosis separately. Lived experience accounts, empirical studies and theoretical models all attest to the interplay between post-traumatic stress and psychosis, meaning that therapies which do not consider the impact of the other may be lacking, potentially detrimentally impacting engagement, adherence, and outcomes [see (31–33)]. For example, a person may hear the voice of a perpetrator, experience sensations of being assaulted and have flashbacks of past attacks, which exacerbate each other. A sole focus on memory processing could be challenging if the person is struggling with the voice commanding them to harm themselves, and CBTp may be helpful to support the person in feeling able to safely engage with memory work. Conversely, working only with the person’s voices may have limited impact if flashbacks repeatedly reinforce the distressing aspects of voice hearing and are not a target for therapy.

To address the issue of how to support the range of trauma effects that may be experienced by people with psychosis, the therapy approach adopted by the PTSp clinic was formulation-based and problem-focused. This was assumed to be a potentially more acceptable approach than delivering diagnosis-based therapies consecutively and is in line with recommendations to integrate exposure within evidence-based therapies for complex populations (8, 34–36). The main therapeutic targets were the psychological mechanisms implicated in fueling post-traumatic stress in people with distressing psychosis, namely emotional regulation, episodic memories and schema or beliefs [see (8, 37)]. A phased, trauma-focused CBT for psychosis (tf-CBTp) approach was used, allowing for flexibly addressing post-traumatic stress or psychosis, dependent on the person’s goals, problem list and obstacles to therapy (38). Nonetheless, memory processing was prioritized when possible, given that elaboration and contextualization of memory is hypothesized to be a key mechanism of change in PTSD, and co-occurring PTSD and psychosis leads to worse outcomes (39–41).

To the best of our knowledge, there has been no audit of a trauma-focused psychological therapy care pathway for people with psychosis who are seeking support for the impact of past trauma. Tong et al. (42, 43) report qualitative and quantitative findings from a case series of people with significant PTSD, dissociation, or trauma-related psychosis in an early intervention in psychosis service in Australia, who received a trauma intervention involving safety, psychoeducation, timeline, and formulation. They found positive evidence of the therapy’s acceptability and benefits, despite symptom exacerbation being common, although the therapy did not include direct memory processing. Keen et al conducted the first case series (*n* = 9) (18) of the feasibility of an integrated cognitive-behavioral therapy protocol, with the sample selected for current PTSD and psychosis symptoms from referrals to a specialist NHS psychological therapy for psychosis clinic (44). Therapy was found to be safe, feasible and acceptable, with promising clinical outcomes. This study provides encouraging evidence that trauma therapies for psychosis can be implemented in an NHS setting. However, the study did not report on referral pathways, which may inform future service developments, specifically the proportion of referrals with significant past trauma for whom tf-CBTp or CBTp was indicated. Further, lived experience perspectives were not explored in detail, limiting the understanding of how therapy provision and delivery was experienced in routine care, particularly memory processing (25). This audit therefore aims to investigate the feasibility, safety and effectiveness of the implementation of trauma-focused cognitive-behavioral therapy for psychosis (tf-CBTp) in a routine outpatient setting, with a focus on routine outcome monitoring of PTSD and lived experience perspectives on tf-CBTp.

## Materials and methods

### Design

A mixed methods design was used, including a within-subjects repeated measures design to examine PTSD outcomes and qualitative semi-structured interviews conducted with a subgroup of participants to explore the therapy experience.

### Service setting

The PTSp clinic was based in one of four geographical boroughs of the South London & Maudsley NHS Foundation Trust, which is culturally diverse and characterized by high levels of deprivation. The clinic’s remit was to provide specialist psychological assessment for people with psychosis who report being significantly affected by past traumatic events and, where indicated, trauma-focused therapy. The clinic had a small, part-time team, led by the first author who specializes in trauma therapies for psychosis (AH, female, White British), with input from a rotation of trainee clinical psychologists and assistant psychologists. All cases were seen by AH or a trainee clinical psychologist (including one White British male and five females; one White British and Black Caribbean, one Black African, two White British, and one White Australian) under the supervision of AH. The audit was approved by the Psychosis Clinical Academic Group, South London & Maudsley NHS Foundation Trust (ref: PSYAUD 16/20).

### Participants

Participants could be referred to the PTSp clinic by multidisciplinary clinicians. This audit focuses on 71 referrals received between 2014 and 2018, with tf-CBTp offered to people meeting criteria for clinically significant post-traumatic stress symptoms including re-experiencing. Therapy completers who had finished therapy at the time of qualitative data collection were invited to participate in the interview study. This resulted in a subgroup of tf-CBTp completors (*n* = 6) consenting to be interviewed about their experience of therapy and giving informed consent for anonymized quotes to be used. This small sample size is acceptable given that this is the first audit of experiences of tf-CBTp in routine care, and therefore has strong “information power” with the interviewees uniquely place to give insights regarding implementation (45).

### Therapy

As previously described, the therapy integrated assessment, formulation, and intervention strategies from tf-CBT and CBTp within a phased tf-CBTp framework, see (32, 37–39). The therapy phases were delivered flexibly, and moved between iteratively dependent on the person’s problems, goals, progress, and preferences. As is emphasized in CBTp, developing a collaborative and safe therapeutic relationship was essential (46), with shared decision making employed throughout. Trust and control were a particular focus, given how these are violated by interpersonal trauma and healing requires trustworthy and empowering relationships (47). The therapy phases are briefly outlined below.

#### Phase one: Assessment, formulation, goal setting and psychoeducation

Assessment was guided by a tf-CBTp formulation model (32, 37). Following a semi-structured interview to explore a person’s hopes for therapy and view of their current problems, trauma history and post-traumatic stress problems were assessed using standardized measures. Formulation involved collaboratively developing a personalized understanding of problems, which was used to guide therapy planning. This included consideration of how memories of past events may be interacting with beliefs and emotion regulation to give rise to distressing experiences (including intrusions related to the self, perception, and memory), explanations (which may manifest as unusual or delusional beliefs), and responses (in cognitive, affective, somatic, behavioral, and interpersonal domains), which paradoxically maintain each other and present obstacles to valued goals. Formulation modality was adapted to the person’s needs and preferences, and could include diagrams, letters, lists and verbal discussion as a means of developing a shared understanding of target problems. As in CBT, formulation was iteratively developed over the course of therapy, such as adding insights from a developmental perspective (e.g., that voices may mirror a belief that ‘I’m worthless’ which developed following bullying) or to target novel maintenance cycles (e.g., the role of hypervigilance and avoidance in maintaining worries about harm from others). Psychoeducation included normalization and validation of common effects of trauma (e.g., intrusive memories, dissociation, hallucinatory experiences and paranoia) using the person’s preferred language for describing their difficulties. Psychoeducation was helpful in providing alternative ways of understanding experiences, laying the foundation for later evaluation of trauma-related beliefs (e.g., a person who believes that feeling ‘detached’ is a sign they are ‘defective’ may find it useful to learn that this is a common, protective response to traumatic events).

#### Phase two: Memory work (contextualizing and elaborating memories)

As outlined above, trauma memory work was central to the therapy, and prioritized where possible. Given the commonalities between different types of memory work approaches such as PE, EMDR, NET, Imagery Rescripting, stimulus discrimination and Reliving with Restructuring (48), any type of memory technique could be used, based on the formulation and shared decision making. Memory work involved: providing a rationale based on the person’s formulation, identifying events that would be targeted (using timelines, hierarchies, belief floatbacks and/or affect bridges), developing an in-session and post-session safety plan (including strategies for managing any obstacles to facing memories), exposure to memories and/or strategies to modify memory content, meanings or interactions, debriefing, and monitoring of impact (e.g., on emotions, meanings, memories, hallucinatory experiences, avoidance and hyperarousal). Memory work aimed to reduce the intensity (e.g., distress, controllability, frequency, meaning) of trauma memory intrusions, elaborating and contextualizing them as past events, including integration of any updating information (using imaginal and cognitive techniques) to develop more helpful trauma-related beliefs and reduce their problematic impact on daily life. Processing memories also served to support people in reducing their avoidance of trauma reminders and associated arousal and dissociative detachment. Regardless of the specific memory approach used, transient increases in intrusions and distress were expected as avoidance reduced and engagement with emotionally laden memories increased. Adverse reactions were closely monitored during therapy and in supervision, with additional support and strategies provided as needed. Given the challenges involved in memory work, close attention was paid to informed consent. A balance was struck between not unhelpfully colluding with avoidance whilst also ensuring that people were empowered to make decisions about whether to commence and continue with memory work, including consideration of alternative therapeutic targets as needed (e.g., managing emotions, coping with re-experiencing, social inclusion).

#### Phase three: Working with beliefs, emotional regulation, appraisals, and emotional, cognitive, behavioral, and interpersonal responses

This phase of the work could draw on a range of strategies from second [see (28, 49)] and third wave CBT for psychosis [see (50)]. This phase targeted problems (linked to therapy goals) that had not been a focus during memory work, or difficulties that persisted following memory work. This usually involved revisiting phase 1, with further assessment, formulation, and support to learn about common trauma effects. People were supported to manage difficult experiences, meanings, behaviors, emotions and relationships, using cognitive, behavioral, and imaginal strategies. There was a focus on making use of new insights or approaches to problems during daily life, with staying well plans developed to continue building on gains made after therapy has ended.

### Measures

#### Trauma and life events checklist [TALE]

The TALE (51) was developed to assess 20 psychologically and physically threatening traumatic event types that may be experienced by people with psychosis, including attachment-disrupting events, psychosis-related traumas, and neglect. Respondents are asked to report whether they experienced an event type, with follow-up questions about frequency and age of occurrence also included. If at least one type of event is endorsed, respondents are asked if events that ended at least a month ago are continuing to affect them (and if so, which are affecting them the most), to identify the relevant past experiences on which to anchor the assessment of post-traumatic stress (subsequently defined as the ‘index’ trauma). The TALE was iteratively developed during the audit period, and therefore an earlier, shorter version of the measure was completed by a minority of respondents.

#### Post-traumatic stress disorder checklist, versions 4 and 5 [PCL-4 and PCL-5]

Both measures assess PTSD symptom severity in relation to an identified past index (‘current worst’) traumatic event(s). The PCL-4 (52) was initially used, with the PCL-5 (53) being introduced when this new updated version was released. The PCL-4 is a 17-item questionnaire with responses ranging from 1 to 5 (1 “not at all” to 5 “extremely”) and assesses the 3 symptom clusters of PTSD as operationalized by DSM IV. Both total severity scores and symptom cluster totals can be calculated. For the former, values range from 17 to 85, with higher scores indicating greater severity. A total score of 44 or above was used to indicate clinically significant PTSD. The PCL-5 is a 20-item questionnaire with the response scale ranging from 0 to 4 (0 “not at all” to 4 “extremely”) and assesses the four symptom clusters of PTSD as operationalized by DSM 5: re-experiencing, avoidance, negative cognitions and mood and hyperarousal. The total symptom severity score ranges from 0 to 80; with higher scores indicating greater severity. Sub-scores can also be calculated for the symptom clusters. A cut-point of 33 and above was employed to suggest clinically significant PTSD. Cluster severity scores were also calculated for both measures. A change score of 10-20 points is considered a clinically meaningful change, on the PCL-4 and 5.

#### Demographic and clinical data

Demographic data and information on referrals, assessments, therapy, and serious adverse events (SAEs) were extracted from medical records by assistant psychologists, trainee clinical psychologists and the clinc lead and stored on the clinic database. In the tf-CBTp sample, SAEs could include deaths, suicide attempts, violent incidents (needing police involvement) and formal complaints about therapy. Any hospital admissions were also recorded.

#### Qualitative data collection

The semi-structured interview schedule was developed through reviewing previous studies of service-user experience of CBT for psychosis and in consultation with experienced therapists. The following topic areas were addressed: (1) the experience of starting therapy, (2) therapist qualities, (3) talking about past difficulties, stressful or traumatic events, (4) therapy endings and outcomes, (5) barriers to treatment, and (6) ideas for future service developments.

### Procedure

Multidisciplinary community psychosis teams were informed of the clinic through leaflets and posters, and introductory talks given. However, due to resource constraints referrals were not regularly sought after the initial launch of the clinic. Following referral, service-users were contacted and offered an assessment with a clinical psychologist and/or trainee clinical psychologist to consider their needs and explore whether tf-CBTp might be suitable for them. During the assessment, the TALE and PCL were completed in an interview format as opposed to self-report. This was partly to reduce burden on the person, and partly to ensure that follow-up questions could be asked as required: for example, to assess whether reported problems (e.g., with sleep or concentration) were associated with the person’s trauma history. The assessment took place over one or two sessions, including feedback and collaborative therapy planning. People meeting criteria for clinically significantly PTSD with re-experiencing were offered tf-CBTp, and the PCL was repeated at the end of therapy to evaluate change in PTSD symptoms. Therapy was provided by the clinical psychologist or trainee clinical psychologist who conducted the initial assessment, with no changes to the therapist once sessions had commenced. People who had completed tf-CBTp were contacted and invited to participate in an interview about their experience of receiving the therapy. Those that indicated an interest in participating were provided with an information sheet about the audit project, given the opportunity to ask questions, and provide informed consent to do the interview if they wished. All interviews were conducted by co-author SG, an MSc student with no prior relationship with the clinic staff or participants, or experience of the implementation of tf-CBTp. The interviews were semi-structured to facilitate exploration of people’s experience of tf-CBTp and were conducted either face to face (five interviews) or on the telephone (one interview).

### Analysis

Clinic referrals and service user demographics are presented descriptively, and clinically significant change reported for PTSD severity on the PCL-4 and 5. Qualitative data from the individual interviews was transcribed and analyzed using thematic analysis following a critical realist framework (54, 55), which assume that engagement with people’s perspectives can generate insights to make sense of subjective experiences (56). Interviews were audio recorded and transcribed verbatim by SG. The transcripts were initially repeatedly reviewed with data patterns identified by EL, followed by line-by-line thematic coding. These were then condensed to generate themes and subthemes, with the final themes and narrative summaries derived by EL and developed in consultation with AH, and consensus achieved. Respondent validation was not possible due to resource constraints during data collection and analysis.

## Results

### Referrals and assessment

**FIGURE 1:**
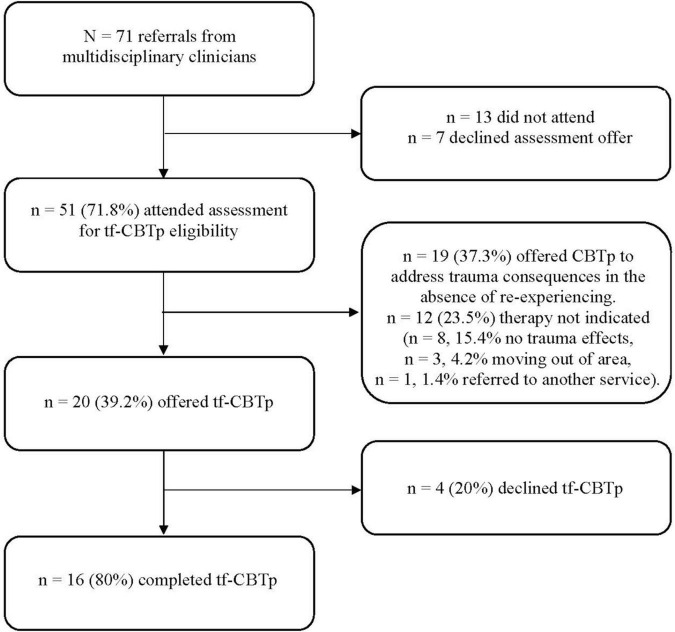
Referral outcomes for the PTSp clinic.

Between 2014 and 2018, 71 people under the care of the borough’s psychosis community teams were referred for an initial assessment (see Figure 1 for referral outcomes). Referrals were made by team psychologists (*n* = 34, 47.9%), care co-ordinators (*n* = 32, 45.1%) and psychiatrists (*n* = 5, 7.0%). The referrals consisted of 39 women (54.6%), with an overall sample mean age of 45.62 (SD = 11.21, age range = 27–64). Their ethnicity was: Black Caribbean (*n* = 18, 25.4%), Black African (*n* = 15, 21.1%), White British (*n* = 22, 30.9%), White Other (*n* = 7, 9.8%), Asian (*n* = 4, 8.4%), South American (*n* = 4, 5.6%) and Dual Heritage (Asian and White British, *n* = 1, 1.4%). Primary diagnosis was: schizophrenia (*n* = 26, 36.6%), unspecified psychosis (*n* = 18, 25.4%), schizoaffective disorder (*n* = 13, 18.3%), bipolar with psychotic features (*n* = 8, 11.3%), depression with psychotic features (*n* = 3, 4.2%), PTSD with psychotic features (*n* = 3, 4.2%), and personality disorder with psychosis (*n* = 2, 2.8%). Following referral, 51 (71.8%) attended for an initial assessment and completed the TALE (or its precursors). Forty-six people (88.4%) reported an index trauma (see Table 1 for trauma type frequencies). The most frequently reported index events were childhood multiple victimization (*n* = 11, 23.9%), followed by childhood sexual abuse (*n* = 9, 19.6%), childhood physical abuse (*n* = 7, 15.2%) and psychosis-related trauma (*n* = 7, 15.2%). People reporting an index trauma were then assessed on the PCL for PTSD symptoms, with 28 (54.9%) scoring above threshold for clinically significant post-traumatic stress [total score > = 44 (PCL 4) or 33 (PCL 5)]. Of these, 20 (39.2%) also met the threshold for re-experiencing, for which memory work was indicated, resulting in an offer of tf-CBTp. Four people (7.8%) declined the offer of tf-CBTp. People who reported post-traumatic stress reactions in the absence of re-experiencing, or who were below the clinically significant threshold (*n* = 19, 37.3%), were offered CBTp to address problems such as trauma-related emotions and beliefs, avoidance, and hyperarousal. Of this group, 14 (27.4%) accepted this offer and 5 (9.8%) subsequently declined. There were 12 people (23.5%) for whom therapy was not indicated, mainly due to a lack of clinically significant trauma consequences (*n* = 8, 15.4%), with a minority moving out of area (*n* = 3, 4.2%) or being referred on to a non-psychosis service (*n* = 1, 1.4%).

**TABLE 1:** Index trauma types in people assessed by the PTSp clinic and the subgroup of tf-CBTp completers.

		Completed assessment (*n* = 51)		Completed tf-CBTp (*n* = 16)	
		n	%	n	%
Childhood	Multiple victimization	11	23.9	4	25
	Sexual abuse	9	19.6	4	25
	Physical abuse	7	15.2	0	0
	Emotional abuse	3	6.5	2	12.5
Adulthood	Multiple victimization	0	0	0	0
	Sexual abuse	1	2.2	1	6.3
	Physical abuse	2	4.4	2	12.5
	Emotional abuse	3	6.5	1	6.3
	War/conflict	2	4.4	2	12.5
	Accident	1	2.2	0	0
	Psychosis-related trauma	7	15.2	0	0

### Trauma-focused cognitive-behavioral therapy for psychosis and post-traumatic stress disorder outcomes

The tf-CBTp group (*n* = 16) was 50% female, with a mean age of 45.70 years, (SD = 11.15; range 30–60). Their ethnicity was: Black Caribbean (*n* = 4, 25.0%), Black African (*n* = 3, 18.8%), White British (*n* = 3, 18.8%), Asian (*n* = 3, 18.8%), White Other (*n* = 2, 12.5%) and Dual Heritage (Asian and White British, *n* = 1, 6.3%). Primary diagnosis was: schizophrenia (*n* = 6, 37.5%), unspecified psychosis (*n* = 4, 25.0%), schizoaffective disorder (*n* = 3, 18.8%), PTSD (*n* = 2, 12.5%) and personality disorder with psychosis (*n* = 1, 6.3%). The index traumas most reported were multiple childhood victimization (*n* = 4, 25%) and child sexual abuse (*n* = 4, 25%). Index traumas occurred prior to the onset of psychosis, with the exception of one person, who experienced an assault after their diagnosis. The mean number of sessions was 17.54 (SD = 17.60, range 12 – 91). Whilst adverse reactions occurred during therapy (e.g., an increase in PTSD symptoms, such as memories or hyperarousal, requiring additional support), often in response to memory processing, there were no SAEs or hospital admissions. Pre- and post- therapy PCL total and change scores are shown in Table 2, together with rates of clinically significant change and clinically significant PTSD following therapy. The mean change score was 34.58 (SD = 17.53), with a change score of 10 – 20 deemed to be of clinical significance (57). Clinically significant change was observed in 68.8% (*n* = 11) people and 37.5% (*n* = 6) no longer met the threshold for clinically significant PTSD. A deterioration in PTSD symptom severity was experienced by 18.3% (n = 3), which was clinically significant for 12.5% (*n* = 2). In both instances, PTSD symptom severity had improved during therapy although this was not sustained, with deterioration associated with an actual or threatened re-occurrence of their index trauma(s).

**TABLE 2:** PCL (PTSD severity) pre and post tf-CBTp, and number of sessions (*N* = 16).

P/total*	Pre-therapy PCL total	Post-therapy PCL total	Change score	Post-therapy clinically significant change	Post-therapy clinically significant PTSD	No of sessions
1	61	31	–30	Y	Y	91
2	62	24	–38	Y	N	12
3	46	50	+4	N	Y	12
4	55	8	–47	Y	N	17
5	60	0	–60	Y	N	12
6	48	69	+21	N	Y	17
7	55	34	–21	Y	Y	20
8	54	48	–6	N	Y	12
9	43	37	–6	N	Y	13
10	43	9	–34	Y	N	15
11	33	7	–26	Y	N	15
12	47	13	–34	Y	N	18
13	63	49	–14	Y	Y	14
14	64	79	+15	N	Y	30
15	62	43	–19	Y	Y	16
16	75	48	–27	Y	Y	52
Mean/% (SD)	45.43 (19.15)	34.31 (23.02)	–20.13 (21.82)	68.8	62.5	17.54 (17.60)

*PCL-5 was completed by all apart from P3 who completed PCL-4.

### Therapy sample: Interviews

The demographics for the interview subsample are shown in Table 3 which indicated that they were representative of the wider therapy sample. Interviews lasted between 50 and 80 min. Findings were organized into four themes: (1) Perseverance, (2) Establishing safety, (3) The challenges of therapy, and (4) Rebuilding one’s life after trauma. These are presented below with corresponding sub-themes and illustrated with verbatim pseudonymized quotes.

**TABLE 3:** Interview sample participant characteristics (*N* = 6).

	n (%)
***Gender***	
Female	4 (67)
Male	2 (33)
***Age range***	
20–29 years	1 (17)
30–39 years	2 (33)
40–49 years	2 (33)
50–59 years	1 (17)
***Ethnicity***	
Black African	1 (17)
Black Caribbean	2 (33)
Dual heritage (Asian and European)	1 (17)
White British	2 (33)
***Types of traumatic index events***	
Adulthood emotional abuse	2 (33)
Adulthood physical abuse	3 (50)
Adulthood sexual abuse	3 (50)
Childhood emotional abuse	4 (67)
Childhood physical abuse	4 (67)
Childhood physical and emotional neglect	2 (33)
Childhood sexual abuse	5 (83)
Racial discrimination	1 (17)

#### Perseverance

The first theme reflected the endurance shown by participants in both accessing, and engaging with, tf-CBTp.

##### Finding suitable help

All six participants shared reflections on the value of specialized support for trauma survivors. For Lisa, the desire for such help was almost existential in its intensity: “*I just wanted to feel more confident about just being human really*.” Ini, in turn, described how limited recovery potential could be without it:

…*this type of experience obviously needs aftercare as well. You can’t just have something happen to you and leave it, it’s not just going to go away, your feelings, views, emotions [are] not going to go away.*”

Nevertheless, there could sometimes be a period of struggle before gaining access to trauma-informed care. In some cases, this resulted from distress being either medicalized or misunderstood; Salma, for example, described how her psychosis diagnosis was a barrier to psychological therapy, while Don relayed how his difficulties were “*overlooked for some period of time*” and considered to be “*just depression*”. Lisa, who had received 11 diagnoses since 2001, likewise described invalidating responses to her distress wherein trauma symptoms were framed as psychiatric disease: “*I don’t believe that, you know, there’s some kind of very rare brain disorder or anything. I know it’s because of my childhood.*”

In this regard the limitations of medication were also noted by some participants, including its inability to get to the “*root*” of the “*real*” problem (Don): “*Tablets don’t cure abuse, neglect [*…*]. With [Therapist] was the first time it was explored and talked through*” (Salma). However, access to therapy likewise did not guarantee relief if practitioners were not sufficiently trauma-informed. For Lisa, this involved a previous therapist who minimized and disbelieved her account of childhood abuse, while Carl described earlier therapy that felt “*empty*”, “*[not] constructive*” and failed to provide specific trauma-focused techniques.

##### Stamina for the therapeutic process

Having withstood the wait to receive help, participants exhibited additional commitment and courage to engage with a process that at times could be extremely demanding. Receiving tf-CBTp could be “*draining*” (Ini), “*hard*” (Don, Lisa), “*overwhelming*” (Salma), “*disturbing*” (Jean) and “*mental work*” (Carl), in which positive gains could still come at a cost: “*It hurt but it helped*” (Lisa).

However, despite such challenges, a consistent theme amongst participants was a willingness to persist to overcome them. In part, this process could be facilitated somewhat naturally over time; for example, as comfort and familiarity with one’s therapist increased, as the process of disclosure and self-expression gradually grew easier, and/or as the rationale for different techniques began to clarify and make more sense. However, participants also described ways they had proactively adapted to a grueling process. Approaches used to make therapy as tolerable as possible included the use of in-session breaks, compartmentalizing session content from everyday life, planning and getting support for getting to and from therapy sessions and a gradual implementation of techniques and change strategies (described by Don as “*baby steps*”) to avoid growing overwhelmed*:* “*it took us slow progress but we done it [*…*] we went out and faced my fears”* (Jean).

In terms of the latter, dedication to therapy could also reflect an intuitive wisdom that healing in the aftermath of trauma was an arduous and ongoing process which could not occur immediately. For Carl, this included recognizing the value of incremental change (“*getting my foot on the ladder, you know, and then my slow climb starts from here*”) while Don described his awareness of therapy not being an “*overnight*” solution, with long-term patience and determination also required. In turn, merely performing certain actions did not appear to be sufficient on their own, instead also requiring an intrinsic sense of commitment:

*Some days it’s hard to get there, but you just have to push yourself and do it, and then over time [*…*] you can see the difference, you gradually can do a little bit more, things are not as bad as they were, over time it just fades [*…*] feelings seem to suppress [*…*] the bad things are not your main focus. [*…*] [I]t’s not a quick fix you’ve got to just, persevere and continue on.*

#### Establishing safety

The second theme reflected relational and intrapsychic factors that empowered participants to make use of, and benefit from, their access to tf-CBTp.

##### Therapeutic alliance

When relating their experiences of tf-CBTp, all participants referred to the varying ways in which their relationships with their therapists had been a source of guidance and solidarity. Therapists demonstrated numerous personal qualities which made it easier to engage with them, including such factors as empathy, courtesy, patience, kindness, perseverance, responsiveness to client experience, instilling a sense of trust, maturity and professionalism, being sympathetic, showing reassurance, a calming demeanor, a willingness to listen and understand, being non-judgmental, and knowing how to “*get on your level*” (Carl). Salma provided the following account of how her aspirations for a caring ally were ultimately fulfiled:

*I just had hope that this person would be kind, gentle, empathetic and would not pathologize things. And although I have a broken brain, I’m living in a broken world and not everything rests on my shoulders. Recovery is not just my responsibility. [My therapist turned out to be] calm, friendly, and the first thing I noticed about [her] was that she normalized things for me, she admitted that no one is free of making mistakes, she wasn’t demeaning, she listened, and she understood, and she didn’t say she knew things when she didn’t. I felt like she fought my corner and had my back. I did very much so feel heard by her.*

In addition to such interpersonal factors, participants also alluded to specific clinical approaches and strategies employed by their therapists that were valued as part of tf-CBtp delivery (see Box 1).

**Box 1 Therapist approaches and strategies that were valued during tf-CBTp.**

A willingness to be thorough and discuss issues in detail

Encouraging overcoming the reservation to describe painful emotion

Encouragement to proactively engage with recovery

Explaining therapeutic rationales in a way that made sense

Helping to address feelings of shame and self-blame

Helping to build confidence by practicing coping techniques in-session

Helping to plan for challenging situations/encounters in advance

Helping to re-evaluate, explore and/or confront fears

Helping to recognize achievements and attributes

Normalizing experiences

Personalizing the treatment protocol to suit preferred pace and needs

Providing in-person support during behavioral experiments

Respecting boundaries for disclosing distressing material

Supporting attempts to socialize and re-engage with enjoyable activities

Using relatable metaphors to make clinical concepts more understandable

Validating client accounts of racism and homophobia within mental health services.

In this respect, the ability to engage with such work could gradually be bolstered by participants’ growing sense of comfort with their therapists. For example, Salma referred to how her therapist personalized their sessions (“*She brought it into my world rather than in a model*”) and, while also noting the power imbalance intrinsic to therapy (“*It’s hard for your life to be open and theirs not*”) further described how her therapist ultimately became a partner in the healing process:

*I found it difficult at first. I thought it would be overwhelming, and it was at times, but [she] was very good at containing it. By talking through any worries, she would hold my hand at times and say: ‘this is normal, this is to be expected and this will get better, and if it doesn’t let me know.*

Taken together, this combination of personal and professional attributes could have a profound effect. Carl, for example, described how his therapist’s “*fantastic way of helping*” was able to “*pull me up [*…*] took me out the dump*” and help discover a source of wisdom and healing which he deemed “*the golden egg*.” Don likewise described therapy as “*like a godsend*” wherein *“this person is sent, because they listen, and they understand, and they help you in different ways; they analyze the problem in different ways that you can understand it.”*

##### Coping strategies

A concurrent feature of safety for many participants was finding ways to manage and mitigate negative emotions, either by utilizing pre-existing strategies or applying ones specifically taught during therapy. For Ini, one such helpful technique was learning breathing exercises which, while not a panacea, she discovered could be applied more holistically than previously realized: “*Where people said that, [*…*] ‘do breathing techniques it helps the body,’ but I didn’t know it actually helps the person [themselves].*” Don likewise mentioned the support and solidarity gained from attending peer support groups, while Jean referred to learning to disregard voice commands, Lisa to listening to music to cope with distress, and Carl to using imagery to gain control over traumatic memories. For him, acquiring “*techniques and rituals*” was the most notable part of tf-CBTp (“*that’s been fantastic, you know I’ve built up a good strong tool kit now*”).

Nevertheless, and despite such benefits, participants also noted that coping strategies were not always entirely effective for either preventing or alleviating distress. Instead, they often required patience, practice, persistence, and a degree of experimentation: “*it may not work automatically, the body’s not used to the process, but if I do it all the time it might eventually work*” (Ini). Jean likewise stated that “*I try my best, I get paranoid obviously, but when I get paranoid and the voices start I try different [*…*] techniques*.” As described by Don:

*[Therapy] doesn’t stop the feelings, it like puts them in a little corner [*…*]. You can find distractions and ways of keeping it at bay, that the effects are not great like they were before. [*…*] It doesn’t disappear, it can put its head up from time to time, but if you have got the tools to put it back in then it goes back in. So that’s what the psychology did [*…*] they give you the tools to deal with the thing.*

In this respect, some participants also related the benefits of re-evaluating previous coping strategies. Carl, for example, described how he had stopped using alcohol as a way to manage emotion (something that occurred prior to receiving tf-CBTp) and that while he had previously employed self-injury to cope, this was no longer something he saw as helpful: “*It’s silly because when you mark yourself you can’t clear it [*…*] I’ve just come through the pain and just accept[ed] it.*” In turn, Salma reflected on how she no longer used social isolation to cope with paranoid feelings: “*Rather than withdrawing, I learnt to challenge thinking and do reality checking with people.*”

#### The challenges of therapy

The third theme reflected the various struggles and constraints experienced throughout the process of engaging with tf-CBTp both in terms of its structure and content.

##### Systemic issues

The first set of difficulties described by participants referred to therapy delivery; specifically, the inherent, organizational limitations of the services within which it was provided. For two participants, this was reflected by a sense of time pressure. Ini, for example, felt that an hour a week was insufficient: “*the time would go really quick, so I had to hurry up and discuss what we had to discuss*.” For her, the opportunity to “*just have someone to hear what I’ve got to say*” was valued, and while she ultimately felt she had “*the time to say what I wanted*”, also noted that “*having more time to talk*” would have been preferred *via* longer and/or more frequent sessions. Lisa, in turn, also summarized the inevitable confines of time-limited therapy: “*I think for me to have gone through at least 20 years of being abused and suffering the consequences, I don’t think 18 months would be enough.”*

Although participants described positive therapeutic relationships, the potential problems of being unable to choose one’s own therapist due to insufficient resources was also noted. For Jean this involved an initial preference to work with women (“*I was scared ‘cause he being a man, I don’t trust men*”), whereas Salma felt that therapists should ideally have a similar lived experience to their clients in terms of race and sexuality. For her, a lack of shared identity could inhibit the willingness to fully express her experiences:

*There were certain aspects that I didn’t feel comfortable talking about. For example, I am a person of color and she is white, I’m gay and she’s straight. You don’t understand how it effects your life if you’re not a minority. [*…*] I’ve had trauma around being a person of color and my family were very badly treated. Because the homophobia and racism was a lesser trauma it wasn’t too much of a problem; but if it had been the main trauma it would probably have been. It didn’t stop me trusting her, but whether she would have understood is another matter.*

Other issues included a preference for the benefits of combining individual therapy with group meetings and discomfort with the therapy venue (“*What I liked least was the environment, a busy CMHT. It didn’t feel like a 100% safe space*”: Salma).

##### Emotional burdens and barriers

Despite its perceived benefits, engaging with therapy could also be a source of considerable distress and doubt for participants. Indeed, for Jean, the challenges of discussing trauma were profoundly triggering and left her feeling “*worse*”:

*When we was talking about my [abuser] [*…*] I didn’t find that helpful at all [*…*] I felt really bad, because that night time I heard the bleeding voices and I had the visions as well. Then I had a fit, then I had two panic attacks.*

For this participant, some things remained too painful to discuss and required a shift in focus within the sessions to areas that were less emotionally threatening. When reflecting on her therapist’s initial encouragement to discuss such “*disturbing*” events, Jean concluded that the process had been partly beneficial (“*it wasn’t 100% but it was quite helpful*”) yet ultimately untenable due to the turmoil it created. In this case, the therapist’s responsiveness enabled the alliance to be preserved, yet also left Jean feeling bereft when she was discharged: “*I felt as if I lost a good friend*.” Despite some of the gains from tf-CBTp sessions, she ultimately noted that post-therapy her distress levels could still become overwhelming, with trauma continuing to trigger nightmares, voices, and incidents of self-harm.

Some similar themes were also expressed by Ini, both in terms of loss at therapy’s conclusion (“*[I] didn’t really want for these sessions to end, I got so used to them; routine, you know*”) and concerns that talking about trauma could make her lose control (“*some people start lashing out [*…*] it could be the emotion comes out in different way [*…*] [A]ll sorts of stuff could happen*”). These reservations did not deter her from confiding in her therapist, yet the process was still “*stressful*” and “*straining*”:

*[I]t’s like it puts me back in a situation I don’t ever want to be back in again. It’s not a nice feeling. It’s a good feeling to release that energy, but at same time it was negative.*

Such disclosure, in turn, did not fully bring relief, with Ini relating how her therapist’s support had helped alleviate feelings of self-blame without entirely resolving them: “*I still feel that it was my fault still, but at the same time I don’t [*…*] [I]t’s like 50/50*.” For Lisa, living with persistent distress in the aftermath of trauma was likewise something therapeutic dialogs felt insufficient to address: “*I wasn’t sure that talking about it would help because I talk about it all day by myself, at home, some of my voices, still I cry and I’m still suicidal and I still cut sometimes*.” In turn, an inability to express the extent of what had happened to her ultimately became a barrier to progress that felt insurmountable:

*I don’t want to say [it’s] my fault. Although I think If I could have been less embarrassed and just speak and just say [*…*] how I really felt, and I was honest and open about what I had been through. But I didn’t talk much about the consequence and that’s; it’s damaging.*

An additional barrier for Lisa was a sense of cultural and ethnic alienation in the aftermath of trauma which was something she felt unable to articulate in-session (“*I don’t know how [*…*] [Therapist] would have responded to that*”), a reservation compounded by pervasive feelings of shame: “*I felt embarrassed I didn’t want to be judged*.” In the event of future therapy, she felt she would be more inclined to “*say what I want to say get everything out*”, stating that “*therapy can’t be successful if you’ve still got a lot of baggage [*…*] locked up inside of you*.” However, when reflecting on the process, she also noted how such struggles were something therapists should be more proactive in encouraging clients to overcome; namely by reiterating the importance of expressing oneself freely and openly:

*[M]aybe like every session, maybe at the beginning and at the end, tell them, you know, the importance of speaking about everything, get everything out regardless. Literally everything, regardless of if you think you’re gonna be judged or you think it’s gonna be embarrassing, if you think you’re gonna look really bad. You know, just get everything out.*

#### Rebuilding one’s life after trauma

The final theme contained participants’ thoughts on how tf-CBTp could provide possibilities to envisage a more peaceful future relative to the pain of the past.

##### Expression and exploration

In various ways, therapy, including memory processing, could be an opportunity to articulate, process and/or begin to make sense of one’s experiences. For some participants, this included an opportunity for emotional expression, which Ini described as a form of catharsis: “*I was crying a lot, so some of my pain was shedding*.” For her, releasing such emotional “*baggage*” was grueling yet also something worth striving for: “*all the bad emotions are supposed to drain from the person [*…*] when it’s stuck it’s negative, it’s not positive*.” As such, she felt that “*talking did a lot for me”* on the grounds that *“the situation was really bad, and the emotion was building, I obviously had to release that emotion*.” Carl, in turn, related how discussing trauma was “*painful, extremely agonizing*” and that “*I cried my eyes out*”, yet likewise noted it could ultimately bring relief: “*you just gotta go there you know, just don’t be afraid [*…*] you gotta talk, there’s no good holding it in, it just eats you away*.” A similar point was also made by Don, who noted the groundwork provided in therapy for navigating emotional pain could help guide subsequent recovery:

*I think that the basis of that [trying new activities] is therapy, really, “cause you have to dissect all these feelings first before you can move to another level. [*…*] I think the therapy has to come first, because to understand your feelings and to be able to manage them, I don’t think other things outside of that will work. [*…*] It’s like the foundation isn’t it?”*

Trauma-focused CBT for psychosis (tf-CBTp) could also be a chance to gain new knowledge and perspectives on one’s suffering, although this was something that could take considerable time. For Salma, therapy initially “*didn’t seem like it was doing anything*” and it was only “*in the last few months [I] can see how its helped and has been life changing. All the jigsaw pieces started to fit together*.” For her, such expression could bring a sense of insight and a chance to reclaim her own narrative: “*The advice I would give is to let your pain talk. Give it a chance to talk, don’t suppress it. Listen to your voices*, *it’s not the truth, it’s been written by other people*.” For Carl, who needed support with “*getting my mind back*” therapy was similarly “*complex at first*”, yet ultimately became a way to organize his mental “*filing cabinet*” wherein “*one by one*” issues could be sorted and stored:

…*when you work on yourself and you get everything corrected, throw out all the negative; and when you got the facts there, you can only put it in when you got the facts, you know, and then you put it in the filing cabinet up in the top draw, put it in, shut it.*

Don similarly related how therapy “*gives you an insight into why you feel like this*” wherein the initial tumult of the sessions could gradually give way to a growing sense of clarity:

…*in the beginning it was a little bit like a tumble dryer; it was making sense, but not making sense, and then in the middle things began to, I could see a picture, and then from that on the picture become clearer. [*…*] I could make sense of feelings I was having, why I felt like that, the things I was doing. Realizing that because of my condition it was just a natural reaction. But at the time I didn’t understand that.*

For Lisa, working with her inner child was “*probably one of the best, or the best, [*…*] psychological therapies I’ve had*.” Bearing witness to her own defenselessness at that age contradicted previous grooming by her abuser and, while provoking feelings of sadness, was “*very helpful*” and made her feel “*like somebody cared, you know like I was [*…*] not me, somebody else, and I just saw vulnerability.*” In turn, other therapy components also provided opportunities for self-expression and to have another person bear witness to her experience.

##### Hope and healing

Despite the challenges experienced during therapy, five participants said they would recommend it to other trauma survivors and one (Ini) stated she “*probably*” would. Correspondingly, several descriptions were provided of ways in which tf-CBTp had the potential to help envisage more positive long-term outcomes, even if these could not be realized immediately. Lisa, for example, noted that she was “*completely social[ly] withdrawn*” at the start of therapy and that socializing was “a lot easier now” despite her continuing struggles: “*I mean it feels maybe 5% willing to try to socialize, but before the day I met [therapist] it was literally 0*.” Carl, who felt “*pleased and triumphant*,” at his progress and “*that I’ve come a long way*” similarly felt that future gains could take time: “*I can’t learn something straight away because it don’t happen that way [*…*] but I’m getting there and I’m working on that*.” Don, in turn, noted that while he still struggled with feeling safe and would sometimes avoid leaving the house, engaging with therapy had helped to develop a growing sense of security for being in the world:

*I can go out, it built my confidence up. My fear, I see fear in a different way. And I see people in a different way. Because I thought all people are bad, they’re all demons, that’s what I thought; they were demons out there. They’re not good, their intentions are bad. This is one person and it’s not the whole world. Yeah, to learn that every time you go outside that something bad is not going to happen, cause that’s how I thought about it.*

For other participants, therapy had provided opportunities to begin changing the way they related to their past experiences. Carl, for example, revealed that there had been a notable shift in the capacity of his memories to devastate him, as well as a certain sense of closure. Prior to therapy, he “*was petrified*” to discuss his trauma, yet reflected that such fears had since receded: “*it’s all cleared up, it’s brand spanking new now, I can talk about it and it don’t affect me, it don’t send me in a twist, it don’t make me all over the place [*…*] it’s gone it’s finished*.”

For Don, who felt that “*I’m proof you come out the other end*” of trauma, tf-CBTp had been “*lifesaving*” and a means of momentum toward recovery: “*it was a pathway for me, so it was only going forward it wasn’t going back, so that’s what I needed and that’s what happened*.” While reiterating that this process was a long-term endeavor, he also provided the following example of striving to move beyond the pain of the past:

…*you have to work from within yourself, you have to just be determined. If I’m just going to wither away in a corner then I’m giving into [perpetrator] [*…*] So you’ve just got to be as strong as possible. Hope I think is just, hope is the word. Try and look forward not back. I’m still trying to get there I’m working on it. [*…*] [E]very day brings its challenges but it’s just to try and do your best, if you feel today then okay don’t beat yourself up about it. Get back up again tomorrow and try, not give up I think is the thing, that’s it. It’s hard but in time you get there.*

In terms of reclaiming a sense of one’s own identify beyond trauma, Salma also described the process of looking toward a more positive future:

*It has made a huge difference in my life. I feel more at peace and have less negative voices. I feel for the first time I am taking control of my life. Not my past, not my voices, it’s me. [*…*] If I hadn’t had therapy I would have committed suicide, that’s my belief. It has made life not only worth living I can see its beauty that I didn’t see before*.

## Discussion

To the best of our knowledge, this audit is the first mixed methods evaluation of a trauma therapy care pathway for users of NHS community psychosis teams. Overall, the findings demonstrate that implementation of tf-CBTp is feasible and safe, with promising indications of effectiveness. Despite limited recruitment resources, there was a consistent rate of referrals and uptake of assessments was excellent for this population, with nearly three quarters attending. Tf-CBTp (which addresses both “reexperiencing” characteristic of PTSD and distressing psychosis) was indicated for 39.2% of referrals. A similar proportion of referrals were experiencing traumatic stress reactions that did not involve significant re-experiencing and were instead offered CBTp to address trauma-related beliefs, experiences, emotions, and coping strategies. Only a minority had no significant effects of trauma at assessment (15.4%) which may have been due to their absence, or people being unwilling or unable to disclose them. However, amongst the latter group, being provided with an opportunity to discuss trauma consequences may have increased the likelihood of sharing in the future, when they felt ready and able to do so. Taken together, the findings suggests that a specialist PTSp care pathway can support the recognition of people with psychosis who are significantly affected by past trauma, and that comprehensive assessment of the phenomenology of post-traumatic stress can assist appropriate access to evidence-based therapies. As highlighted by the qualitative interviews, people reported that access to trauma-focused therapy required perseverance, sometimes with considerable delays. Whilst Chadwick and Billings (58) found that staff anxieties about implementation are common, this audit demonstrates the feasibility of a specialist care pathway and supports improved provision of trauma therapies in NHS psychosis services.

The majority (80%) of people who were offered tf-CBTp completed therapy, suggesting that the approach was viewed as appropriate for their problems. Clinically significant changes in PTSD symptom severity were found for two thirds of people, with just over a third no longer meeting criteria for PTSD. This was supported by the thematic analysis, with participants reflecting on how the therapy supported expression and understanding, alongside providing possibilities for a more peaceful future relative to the pain of the past. This indicates that routine care provision of tf-CBTp can deliver the beneficial effects found in research, although it is noted that the effects were more modest than those found in an RCT of EMDR or PE for PTSD in people with a lifetime diagnosis of psychosis (22). This is consistent with the therapy effectiveness literature, where effects are found to reduce outside of an RCT context (24). The results are also comparable to Keen et al. (18), whose case series was likewise conducted in routine care amongst people with current psychosis. They reported that 55.6% of their sample demonstrated reliable change in PTSD symptoms post-therapy, which is in line with the 68.6% of the tf-CBTp subgroup who had clinically significant change in this study.

Although most of the sample experienced benefits, the focus on painful traumatic experiences and their effects meant therapy was often emotionally challenging and burdensome. Whilst there were no SAEs in the audit sample, adverse reactions were anecdotally noted as common, often in response to memory processing. This finding is consistent with other studies investigating the frequency of exacerbation. For example, Burger et al. (59) found that both early and between-session exacerbation was frequent (32.3% and 46.5%) amongst 99 participants who received either PE or EMDR in the van den Berg (22) trial, but that neither were related to drop-out or treatment response. Exacerbation was further explored in a mixed methods analysis by Tong et al. (42) of a case series of trauma-informed therapy in early intervention services. They found that while exacerbation was common and did not negate the potential value of therapy, the importance of being in control, being supported, and feeling emotionally ready facilitated both disclosure and therapy benefits. This corresponds with our interviewees’ descriptions of how engagement with tf-CBTp could be enhanced through the therapeutic alliance and coping strategies to support a sense of safety.

Whilst positive effects were found in the therapy subgroup, it is important to recognize tf-CBTp is not a panacea. The majority still had clinically significant PTSD symptoms at the end of therapy, and the interviews likewise reflected the enduring effects of trauma and ongoing need for support. This included struggling with the time-limited nature of the work, mostly as a result of organizational and resource constraints. The mean length of therapy was 17 sessions, although there was a significant range, with some people being seen by the clinic lead for a longer duration when indicated. Whilst the case with the greatest gains was only seen for 12 sessions, this was largely attributable to the person reevaluating their memories of past trauma as a construction of their anxieties about interpersonal relationships, as opposed to being a result of experienced abuse. Therapy duration for most cases was limited by the clinic’s trainee clinical psychologists being on placement for six months. NICE recommend a minimum of 16 sessions over six months or longer for psychosis, whereas therapy lasting 12 months plus boosters is recommended for complex PTSD where there are multiple or chronic traumas (26, 60). Therefore, an extended duration and/or higher frequency of sessions may have resulted in greater benefits, as has been found for CBTp and in other groups with high rates of traumatization, such as people with a diagnosis of borderline personality disorder (61, 62). Provision of a broader range of trauma therapies is also likely to improve therapy uptake, engagement, and outcomes, and it would be preferable for people to be offered a choice of therapeutic interventions. For example, dialogical approaches, such as Talking with Voices and AVATAR therapy, show promise in supporting people with trauma-related voice hearing, and warrant further investigation (63–65).

A lack of workforce diversity was another systemic factor relevant to the experience of tf-CBTp. Encouragingly, the ethnic diversity of referrals and the tf-CBTp subgroup were representative of the local community, in contrast to previous findings indicating that Black people may be less likely to receive CBTp compared to their White counterparts (66). However, two interviewees noted that ideally therapists should have a similar lived experience, in relation to gender, sexuality or ethnicity, and that this lack of shared identity was an obstacle to therapy, particularly at the beginning. Given that marginalized groups, particularly those from minoritized ethnicities, are more likely to experience trauma and psychosis due to structural inequalities, there is an acute need to develop a workforce that includes sufficient diversity and culturally sensitive or adapted therapies, deliverable in accessible community settings [see (67, 68) for discussion of these issues in relation to anti-racist practice]. The risk of ongoing victimization was also highlighted by two cases, with psychosocial stressors in the form of actual or threatened reoccurrence of their index traumas leading to a clinically significant deterioration in PTSD, undermining previous gains and thus emphasizing the need for ongoing safety management. Similarly, Brand et al. (69) conducted a qualitative analysis of a case series of PE for voice hearing (16) and recommended monitoring and adaptions to therapy where indicated, after finding the therapy experience was negatively impacted by aversive external circumstances. Social work and safeguarding interventions may be necessary in these cases, further highlighting the value of delivering tf-CBTp in a multidisciplinary setting.

Limitations of this audit study should be noted. First, data collection was constrained by resources (e.g., there was no follow-up assessment) and impacted by the pragmatics of delivering therapy in routine care (e.g., using different versions of the measures). In turn, pre- and post-therapy assessments of other trauma consequences and psychosis would have been beneficial as this was only done for PTSD symptom severity. Further, robust conclusions cannot be drawn about therapy effects on psychosis, as whilst some interviewees commented on these experiences they were not explored routinely. Standardized questionnaires of complex PTSD (cPTSD) were also unavailable at the time of data collection [e.g., the International Trauma Questionnaire, ITQ, (70)] although it is noted that the defining features of cPTSD (i.e., negative beliefs about self, relationship problems and difficulties with managing emotions) are captured, to some extent, by the PCL.

Second, while limiting the sample to those willing and able to be referred was appropriate given therapy should only be considered in this context (42), this did inevitably result in a self-selecting sample. However, only a minority of those offered tf-CBTp declined therapy (20%), there were no dropouts once therapy started and the interview subgroup was also based on consecutive therapy completers. The sample size was relatively small, due to the modest staffing of the PTSp clinic, and replication in larger samples and service settings is required. Although the thematic analysis suggests that benefits were linked to the therapy experience, the study design was uncontrolled and it is not possible to conclusively determine if the effects of tf-CBTp are attributable to therapy or time. However, the learning from therapy delivery in this audit has contributed to the therapy protocol for the Study of Trauma & Recovery, which is a currently recruiting multisite RCT of tf-CBTp (71) and will be the first to provide robust evidence of the effectiveness and cost-effectiveness of this therapy compared to treatment as usual, as well as investigating mechanisms of change. The STAR trial will also investigate potential moderators of therapy outcome, such as demographics, trauma history and therapy dose, furthering understanding of potential confounders of therapeutic effects.

In conclusion, we hope that this audit study will provide encouragement to clinicians to implement trauma therapies for psychosis in their local services, in line with the NHS Long-Term Plan (72). As highlighted, this should involve comprehensive assessment of post-traumatic stress (e.g., to determine the suitability of memory processing, as recommended by NICE (27) as well as of traumatic events and their effects using a broad definition). For those offered tf-CBTp, there should be careful and consistent discussion of informed consent, including the likelihood of exacerbation due to memory exposure, and a focus on the therapeutic alliance and coping strategies to support people to make use of therapy. Other trauma therapies can also be considered during collaborative therapy planning, particularly if trauma memories are not an obstacle to the person’s therapy goals, or if people do not feel ready or able to focus on past memories. CBTp is a trauma-informed therapy that can address trauma consequences, yet this may not be done routinely, and we encourage therapists to collaboratively assess, formulate and intervene with trauma-related difficulties where indicated. Clinicians should be aware that novel trauma therapies are being researched and keep up to date with the emerging evidence and access training where possible. Furthermore, local implementation plans should include co-production and co-design, incorporating perspectives of those from marginalized groups to develop accessible therapy care pathways for psychosis.

## Data availability statement

The raw data supporting the conclusions of the article will be made available by the authors upon reasonable request.

## Ethics statement

The studies involving human participants were reviewed and approved by Psychosis Clinical Academic Group, South London & Maudsley NHS Foundation Trust (ref: PSYAUD 16/20). The participants provided their written informed consent to participate in the interview study.

## Author contributions

AH led on the study design, delivered or supervised therapy cases, participated in the acquisition, analysis and interpretation of the data, took the lead for drafting and revising the manuscript, gave final approval of the version to be published, and agreed to be accountable for all aspects of the work. SG contributed to the development of the topic guide, led the qualitative interview data collection, made substantial contributions to drafting and revising, gave final approval of the version to be published, and agreed to be accountable for all aspects of the work. JD contributed to data collection and cleaning of the quantitative data, made substantial contributions to drafting and revising, gave final approval of the version to be published, and agreed to be accountable for all aspects of the work. EL led the thematic analysis and write-up of qualitative interviews, made substantial contributions to drafting and revising, gave final approval of the version to be published, and agreed to be accountable for all aspects of the work. All authors contributed to the article and approved the submitted version.
