# Corrigendum: “It hurt but it helped”: A mixed methods audit of the implementation of trauma-focused cognitive-behavioral therapy for psychosis

**DOI:** 10.3389/fpsyt.2022.1102162

**Published:** 2022-12-08

**Authors:** Amy Hardy, Sophie Good, Jayde Dix, Eleanor Longden

**Affiliations:** ^1^Institute of Psychiatry, Psychology and Neuroscience, King's College London, London, United Kingdom; ^2^South London and Maudsley NHS Foundation Trust, London, United Kingdom; ^3^North East London NHS Foundation Trust, London, United Kingdom; ^4^Psychosis Research Unit, Greater Manchester Mental Health NHS Foundation Trust, Manchester, United Kingdom; ^5^Division of Psychology and Mental Health, School of Health Sciences, Faculty of Biology, Medicine and Health, Manchester Academic Health Science Centre, The University of Manchester, Manchester, United Kingdom; ^6^Complex Trauma and Resilience Research Unit, Greater Manchester Mental Health NHS Foundation Trust, Manchester, United Kingdom

**Keywords:** trauma, psychosis, post-traumatic stress, cognitive-behavioral therapy, trauma therapy

In the published article, there was an error in the **Funding** statement where in the funding source for author “Eleanor Longden” was omitted. The correct **Funding** statement appears below.

